# Post-translational modifications of fibrinogen: implications for clotting, fibrin structure and degradation

**DOI:** 10.1186/s43556-024-00214-x

**Published:** 2024-10-31

**Authors:** Francesca Nencini, Alessandra Bettiol, Flavia Rita Argento, Serena Borghi, Elvira Giurranna, Giacomo Emmi, Domenico Prisco, Niccolò Taddei, Claudia Fiorillo, Matteo Becatti

**Affiliations:** 1 Department of Experimental and Clinical Biomedical Sciences “Mario Serio”, University of Firenze, Firenze, Italy; 2 Department of Experimental and Clinical Medicine, University of Firenze, Firenze, Italy; 3 Department of Medical, Surgical and Health Sciences, University of Trieste, Trieste, Italy

**Keywords:** Fibrin, Fibrinogen, Post-translational modifications, Thrombosis

## Abstract

Fibrinogen, a blood plasma protein with a key role in hemostasis and thrombosis, is highly susceptible to post-translational modifications (PTMs), that significantly influence clot formation, structure, and stability. These PTMs, which include acetylation, amidation, carbamylation, citrullination, dichlorination, glycation, glycosylation, guanidinylation, hydroxylation, homocysteinylation, malonylation, methylation, nitration, oxidation, phosphorylation and sulphation, can alter fibrinogen biochemical properties and affect its functional behavior in coagulation and fibrinolysis. Oxidation and nitration are notably associated with oxidative stress, impacting fibrin fiber formation and promoting the development of more compact and resistant fibrin networks. Glycosylation and glycation contribute to altered fibrinogen structural properties, often resulting in changes in fibrin clot density and susceptibility to lysis, particularly in metabolic disorders like diabetes. Acetylation and phosphorylation, influenced by medications such as aspirin, modulate clot architecture by affecting fiber thickness and clot permeability. Citrullination and homocysteinylation, although less studied, are linked to autoimmune conditions and cardiovascular diseases, respectively, affecting fibrin formation and stability. Understanding these modifications provides insights into the pathophysiology of thrombotic disorders and highlights potential therapeutic targets. This review comprehensively examines the current literature on fibrinogen PTMs, their specific sites, biochemical pathways, and their consequences on fibrin clot architecture, clot formation and clot lysis.

## Introduction

Thrombosis is a leading cause of death worldwide and includes arterial events (myocardial infarction and ischemic stroke) and venous thromboembolism (VTE), that comprises superficial and deep vein thrombosis (SVT and DVT) and pulmonary embolism (PE) [1]. Thrombosis can be triggered by diverse factors such as trauma, non-traumatic insults, or various clinical disorders. Thrombotic events can occur in the whole vascular network, ranging from major arteries to the smallest capillaries, impacting organ and tissue function and structure. Arterial and venous thrombosis are influenced by Virchow’s triad, involving endothelial injury, disturbances in blood flow, and alterations in platelet and plasma constituents favoring thrombosis [2, 3].

Fibrinogen, a large hexameric glycoprotein produced primarily in the liver, plays a crucial role in hemostasis, serving as the precursor to fibrin, the main protein component of blood clots [2, 4–6].

The study of fibrinogen began in the 19th century with Rudolf Virchow’s identification of fibrin as a key element in blood clots. It wasn’t until 1937 that scientists confirmed that proteolytic enzymes could convert fibrinogen into fibrin, establishing the role of limited proteolysis in clot formation [7]. The culmination of these studies came in 1952 when John Ferry proposed that the removal of negatively charged peptides from fibrinogen leads to spontaneous polymerization, forming protofibrils [8]. Subsequent research throughout the 20th century, including advances in electron microscopy and crystallography, revealed the trinodular structure of fibrinogen and its three polypeptide chains culminating in detailed structural and mechanistic insights into how fibrinogen transforms into fibrin. These discoveries laid the foundation for understanding blood clotting and the broader implications of fibrinogen in health and disease [9].

Fibrinogen molecule comprises two sets of three polypeptide chains (Aα, Bβ, and γ) linked by disulfide bonds, forming a complex structure essential for its function in coagulation. Upon vascular injury, fibrinogen is converted by thrombin into fibrin, which polymerizes to form a stable clot, a key step in stopping bleeding [2, 4]. Beyond its role in clot formation, fibrinogen interacts with cell surface receptors, modulating platelet aggregation and linking coagulation with inflammatory pathways [10, 11]. Its concentration and function are tightly regulated, with disorders in fibrinogen levels or structure being associated with both bleeding and thrombotic conditions [12]. Quantitative and qualitative changes in fibrinogen, resulting in a fibrinogen “multiplicity”, can therefore modify fibrin networks and thrombus architecture, with important functional consequences that may underlie the main cardiovascular diseases.

Congenital fibrinogen disorders further illustrate the complexity of fibrinogen’s role in hemostasis and thrombosis. These disorders, such as afibrinogenemia, hypofibrinogenemia, dysfibrinogenemia, and hypodysfibrinogenemia, result from various genetic mutations in the fibrinogen genes (FGA, FGB, and FGG) that lead to altered fibrinogen synthesis, secretion, or function. The genetic diversity within these disorders contributes to a wide range of clinical presentations, from bleeding to thrombotic predispositions, underscoring the significant variability in fibrinogen’s function even among individuals with the same genetic disorder [13, 14]. These congenital disorders underscore the critical importance of understanding fibrinogen’s structural and functional variability, which has profound implications for disease pathogenesis and the development of targeted treatment strategies. In addition to congenital variations, fibrinogen heterogeneity is further enhanced by genetic polymorphisms, alternative mRNA splicing, and a wide range of post-translational modifications (PTMs).

Fibrinogen PTMs, such as phosphorylation, glycosylation, oxidation, and nitration, further modulate its structure and function, influencing clot formation, architecture, and stability. These modifications can be introduced enzymatically or through interactions with reactive species, significantly impacting fibrinogen’s role in coagulation and its interaction with other cellular components. PTMs have been shown to alter fibrinogen’s ability to form clots, affect the mechanical properties of fibrin, and influence susceptibility to fibrinolysis, thereby playing a critical role in various pathological conditions, including cardiovascular diseases, inflammatory states, and metabolic disorders [15–18].

Given the extensive implications of PTMs on fibrinogen’s function, understanding these modifications is crucial for advancing our knowledge of hemostasis and developing targeted therapies for coagulation disorders.

Here, we selected articles based on their relevance, impact, and contribution to understanding the effects of PTMs on fibrinogen and clot formation. Our selection process involved a comprehensive search of the literature using databases such as PubMed, Scopus, and Web of Science. We focused on studies that provided novel insights into the biochemical mechanisms of PTMs and their implications for clot architecture, stability, and lysis. Priority was given to recent publications that offered new data or interpretations not covered in previous reviews. Additionally, we included key foundational studies that have been influential in shaping the current understanding of PTMs’ role in coagulation. This narrative review synthesizes findings from in vitro, ex vivo, and clinical studies, highlighting significant advancements and ongoing debates in the impact of PTMs on fibrinogen’s structure and function, emphasizing their role in coagulation and fibrin clot dynamics. It explores the structural and functional properties of fibrinogen, highlighting its critical role in clot formation and stability. The review delves into the various PTMs that fibrinogen can undergo, detailing the biochemical mechanisms behind these modifications and their effects on fibrinogen structure, fibrin clot architecture, clot formation and dissolution. It underscores the importance of understanding these modifications as they significantly alter fibrinogen’s biochemical properties, influence the mechanical characteristics of fibrin clots and contribute to fibrinogen’s molecular heterogeneity. Furthermore, the review identifies gaps in the current knowledge and suggests future research directions, emphasizing the need for deeper exploration into the diverse roles of PTMs in hemostasis and thrombotic disease management to enhance therapeutic strategies for coagulation disorders.

## Fibrinogen architecture and PTMs

### Implications for clot structure and function

**Fig. 1:**
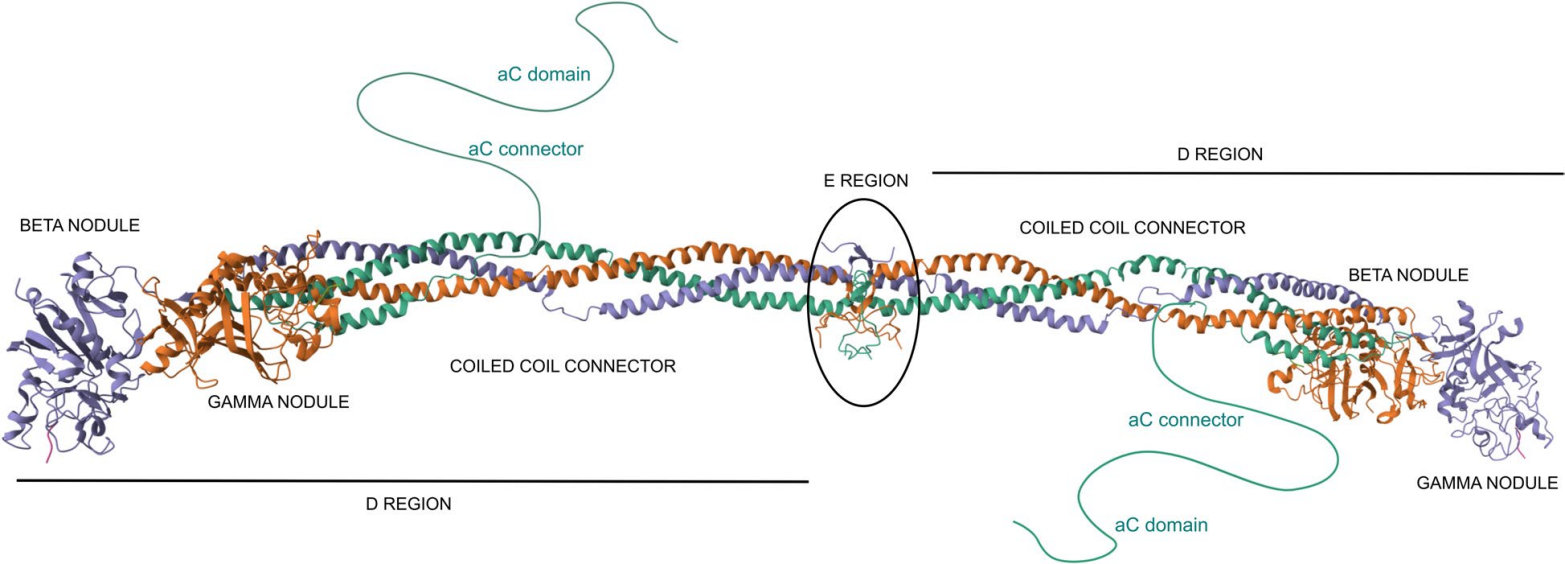
Structure of fibrinogen based on its crystal structure 3GHG (Kollman, ö.M.; Pandi, L.; Sawaya, M.R.; Riley, M.; Doolittle, R.F. Crystal Structure of Human Fibrinogen. Biochemistry 2009, 48, 3877–3886.). Missing parts of the molecule are schematically drawn into the figure. The Aα chain is shown in green, the Bβ chain in orange, and the γ chain in grey-violet

Fibrinogen molecule has a dimeric structure composed of two sets of three polypeptide chains – Aα, Bβ, and γ – consisting of 610, 461 and 411 amino acids, respectively, and connecting a central E region to two outer D regions via coiled-coil connectors. The central E region comprises the N-termini of the polypeptide chains, including fibrinopeptide A (FpA) and fibrinopeptide B (FpB). The distal D regions include the β- and γ-nodules, each with A-, B- and P-domains. A fourth region consists of the αC domains, which are connected to the coiled-coils by the αC connectors. (Fig. 1. 3GHG, PDB DOI: 10.2210/pdb3GHG/pdb). Until recently, the full three-dimensional structure of fibrinogen was elusive, largely due to its high flexibility, which poses challenges for crystallographic analysis.

Mature human Aα chain can be divided into fibrinopeptide A (16 N-terminal amino acids of the Aα chain), that is cleaved out during the conversion of fibrinogen to fibrin, and an α fibrin chain, that remains in the fibrin hexamer. The N-terminal region of the fibrinogen Aα chain is functionally important for fibrin polymerization, but the majority of interactions involve the αC region of fibrinogen. This region makes up two thirds of the Aα chain and contributes approximately to 25% of the mass of fibrinogen. The αC region is crucial for fibrin polymerization, cross-linking, fibrinolysis and interactions with other plasma proteins, that include FXIII, fibrinolytic proteins plasminogen and tPA (tissue-type plasminogen activator), as well as their inhibitors, α2-AP (α2-antiplasmin) and PAI-1 (plasminogen activator inhibitor type 1) [19–21]. In addition to binding plasma proteins, the fibrinogen Aα chain can also interact with integrins on cell surfaces, such as those found on platelets and endothelial cells. The αC-region of fibrinogen has been identified as a crucial area for its interaction with Glycoprotein VI (GPVI), highlighting how the binding of fibrinogen and fibrin to the GPVI receptor on the surface of platelets influences thrombosis [22–25].

Similarly to the Aα chain, Bβ chain comprises fibrinopeptide B (14 N-terminal amino acids), that is cleaved out during conversion to fibrin, and the adjacent fibrin β chain.

The γ chain contains a number of sites that interact with other fibrin(ogen) molecules, clotting factors, growth factors, and integrins. A minor variant of the γ chain, called γ′, arises from alternative processing of the primary mRNA transcript and amounts to approximately 8% of the total γ chain population. It consists of 427 residues and differs from γ chains in that the four C-terminal γ residues, AGDV, are replaced by an anionic sequence of 20 amino acids that includes two sulfated tyrosines. Unlike the main form of fibrinogen, the γ′ chains modulate thrombin and FXIII activity, influence clot architecture, and do not bind to the platelet fibrinogen receptor, αIIbβ3 [26, 27].

During coagulation, fibrinogen is converted to insoluble fibrin through a sequence of thrombin-catalyzed reactions. Thrombin cleaves fibrinopeptides A and B from the Aα and Bβ chains of fibrinogen, revealing α- and β- “knobs.” These exposed knobs fit into corresponding “holes” in the γC and βC regions of the D nodule on neighboring fibrin monomers. This interaction promotes the staggered alignment of fibrin monomers into linear protofibrils. These protofibrils then undergo lateral aggregation, forming thicker fibrin fibers that weave together to create a stable fibrin mesh. This meshwork is vital for stabilizing the blood clot at the site of injury. Cross-linking of the fibrin fibers by factor XIIIa further reinforces the clot, ensuring its resilience to mechanical stress while aiding in wound healing and preventing further blood loss [28–30].

The architecture of fibrin clots characterized by an open porous network, is crucial for their biological function in hemostasis, fibrinolysis, and wound healing, providing distinctive mechanical features. Fibrin clots exhibit viscoelastic behavior, combining reversible elasticity with irreversible plasticity or viscosity. Under challenging conditions like arterial shear, fibrin clots exhibit strain stiffening, where their stiffness increases with higher strain, aiding in damage resistance. Moreover, fibrin clots demonstrate exceptional extensibility and compressibility, allowing them to withstand substantial deformation without breaking [6].

The properties of the fibrin network can be greatly modulated by a wide variety of factors, including multiple mRNA transcripts (generated by initiation of transcription by alternative promoters, differential termination of transcription, alternative mRNA splicing, or genetic recombination), environmental factors, fibrinogen PTMs and pathological conditions [2, 18, 31–35]. These factors can influence fibrin susceptibility to plasmin-induced lysis, potentially creating a fibrin network that is more resistant to lysis and thus increasing the risk of thrombosis. Conversely, they can result in a fibrin clot that is more susceptible to lysis, rendering it weak and unstable, and thereby increasing the risk of bleeding [12, 36].

Among these factors, PTMs exponentially increase the complexity and heterogeneity of fibrinogen and clot structure. PTMs are reversible or irreversible chemical modifications that can be introduced into the protein structure enzymatically or through bonds between amino acid side chains and reactive species such as oxygen, nitrogen, sulfur, carbonyl, selenium, chlorine, or bromine free radicals [37, 38]. These reactions can modify the fibrinogen molecule in numerous ways, such as phosphorylation at specific serine and threonine sites, hydroxylation of proline, sulfation of tyrosine, deamidation of asparagine or glutamine, formation of N-terminal pyroglutamate from glutamine precursors, oxidation of methionine, histidine, and tryptophan residues, nitration of tyrosine, various modifications of cysteine residues, and the formation of dityrosine and carbonyl groups [34, 39, 40].

Physiologically, low levels of PTMs are present in all proteins and influence various protein functions such as activity, localization or interaction with other molecules or cells, as well as key biological processes such as cell differentiation and gene regulation. At high concentrations, however, they have been reported in several diseases such as myocardial infarction, arterial and venous thrombosis, pulmonary embolism, cancer, infections [41–51].

Numerous in vitro and ex vivo studies characterized and assessed the effects of fibrinogen PTMs. Specifically, the extent of PTMs induced in vitro on the fibrinogen molecule depends on the type of reagents, their concentration, and the duration of fibrinogen exposure [52]. Ex vivo, PTMs can occur naturally, in response to certain drugs or pathophysiological conditions. PTMs can involve various sites on the fibrinogen molecule and can lead to altered fibrinogen structure/function and fibrin clot properties.

**Fig. 2:**
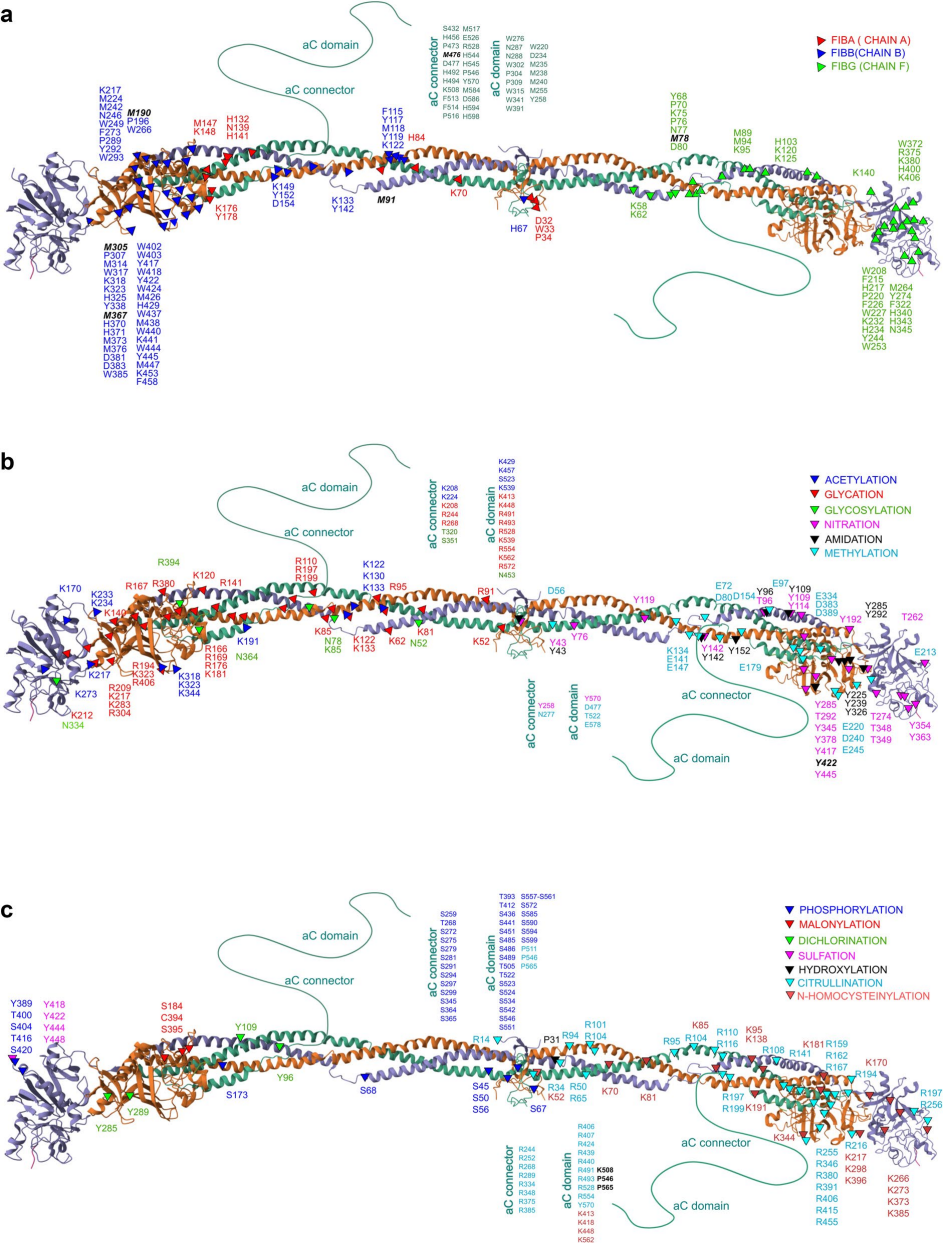
**a** Known sites of oxidation on the fibrinogen chains. **b** and **c** illustrate the specific sites of major post-translational modifications (PTMs) on fibrinogen chains. Each letter-number combination indicates the type of amino acid, represented by its one-letter code (e.g., A for Alanine, R for Arginine, N for Asparagine, D for Aspartic acid, C for Cysteine, Q for Glutamine, E for Glutamic acid, G for Glycine, H for Histidine, I for Isoleucine, L for Leucine, K for Lysine, M for Methionine, F for Phenylalanine, P for Proline, S for Serine, T for Threonine, W for Tryptophan, Y for Tyrosine, V for Valine), and its position within the chain. For clarity, PTMs are shown on only one set of the three fibrinogen polypeptide chains. PTMs that have been specifically identified in the literature as significantly affecting clot formation, fibrinolysis, and key clot properties are emphasized in bold italics. The fibrinogen structure reported is based on NMR model PDB file 3GHG (https://www.rcsb.org/3d-view/3ghg)

While numerous studies have explored the effects of fibrinogen PTMs, only a limited number have specifically investigated site-specific modifications to determine their varying impacts on clot structure and properties. An overview of the known sites of modifications in the fibrinogen chains is provided in Fig. 2a-c.

Weigandt et al. examined the effect of fibrinogen oxidation with hypochlorous acid and found that this treatment preferentially oxidizes specific methionine residues AαM476, BβM367, γ78 on the α, β, and γ chains of molecule [53].

Burney et al. [54] investigated the molecular-level consequences of selective methionine oxidation and reported how oxidation of AαM476 and BβM367 leads to altered fibrin polymerization. Oxidation of AαM476 was also studied by Pederson et al. [55] who reported that this amino acid is necessary for αC domain dimerization and that its oxidation is thought to hinder its ability to polymerize, disrupting the lateral aggregation of protofibrils.

Yurina et al. [56] investigated the effects of very low and moderately low concentrations of HOCl/OCl on the oxidative modifications of fibrinogen and its structure and function. They found that, unlike 25 µM HOCl/OCl, a concentration of 10 µM HOCl/OCl did not impact fibrinogen’s functional activity. Their study demonstrated that several methionine residues—AαMet476, AαMet517, AαMet584, BβMet367, γMet264, and γMet94—identified in fibrinogen exposed to 10 µM HOCl/OCl using the HPLC-MS/MS method, function as reactive oxygen species (ROS) scavengers, playing a crucial antioxidant role. The irreversible conversion of methionines to methionine sulfoxide/sulfone, which occurred in a dose-dependent manner with HOCl/OCl, suggests that fibrinogen’s antioxidant capacity can be significantly depleted, potentially leading to further chemical modifications of essential sites.

To date, oxidative PTMs at various fibrinogen sites (AαM91, AαM476, BβH16, BβM190, BβM305, BβM367, γM78) and nitration at BβT422 have been described in the literature as influencing alterations in clot formation, dissolution, and overall clot properties [32, 47, 57–60]. Specifically, “selective” oxidation at the above listed sites decreases the rate of polymerization and fibrinolysis and results in more dense fibrin clots with thinner fibers which are less permeable. As for “selective” nitration at site BβT422, it increases the rate of clot formation, the stiffness and viscosity of clot as well as the diameter of fibrin fibers, while fibrinolysis is decreased.

 The findings from all studies on each modification are summarized in Table 1, and an overview of the effects of the different PTMs is presented in Table 2.

**Table 1:** Effects of PTMs on fibrinogen function, clot formation and degradation

**Modifications**		**Fibrinogen analysis**					**Clot analysis**				
			**Polymerization kinetics**								
**Author**	**Method**	Fibrinogen polymerization	Lag phase	Max Abs	V max	Fibrinogen Structural Alterations	Fiber Diameter	Stiffness	Permeability	Density	Fibrin Lysis
**Oxidation**											
Roitman et al (2004) [61]	UV-irradiation of fibrinogen	-						-		-	=
Nowak et al (2007) [62]	Fibrinogen + 10 μmol peroxynitrite	=	=	=		Dityr-PC					
Nowak et al (2007) [62]	Fibrinogen + 100-1000 μmol peroxynitrite	-	+	-		Dityr-PC					
Andrades et al (2009) [63]	Bovine fibrinogen or human plasma + 1mM glycolaldehye	-	+	-	-	PC	-				-
Azizova et al (2009) [64]	Fibrinogen + 50-500 μmol FeSO_4_+ 10-250 μmol H_2_O_2_	-	+		-						
Piryazev et al (2009) [65]	Fibrinogen + 50-500 μmol FeSO_4_ H_2_O_2_	-		-							=
Rosenfeld et al (2009) [66]	Fibrinogen + 200-600 nmol ozone	-	+			PC	+				
Tetik et al (2011) [67]	Fibrinogen + 100μM Fe^3+^/ascorbate	-			-	Dityr		-			-
Weigand et al (2012) [53]	Fibrinogen + 50-150 μmol HOCl/g fibrinogen	=					-	-	-	+	-
Stikarova et al (2013) [68]	Fibrinogen + 10mM MDA			-		PC	-			+	
Stikarova et al (2013) [68]	Fibrinogen + 1.25mM NaOCl	-		-		PC	-			+	
Stikarova et al (2013) [68]	Fibrinogen + 100 μmol SIN-1			-		PC	-			+	
Becatti et al (2014) [42]	Fibrinogen + 0.01-1 mM AAPH	-	+	-	-	CD-PC					-
Torbitz et al (2015) [52]	Fibrinogen + 1, 2, 4 mM HOCl	+									
Wang et al (2016) [69]	Fibrinogen + H_2_O_2_					CD-Dityr-IF	-			+	
Wang et al (2018) [70]	Fibrinogen + 0.5 mM H_2_O_2_ + 3 mg/ml Fe_3_O_4_	-				CD-IF	-	-+		+	
Yurina et al (2019) [58]	Fibrinogen + 50, 500 or 1500 μmol HOCl/mg fibrinogen	-		-							
Pederson et al (2019) [55]	Met^476^ unoxidized and oxidized αCdomain dimer						-	-		+	
Gligorijević et al (2020) [71]	Fibrinogen + 5 mM AAPH + DHLA					IF					
Gligorijević et al (2020) [71]	Fibrinogen +2,5 mM AAPH + Resveratrol	-				CD-IF					-
Rosenfeld et al (2021) [72]	Fibrinogen + 25-300 μmol HOCl/mg fibrinogen	-		-		CD			-	+	
Lau et al (2021) [73]	Fibrinogen + 10-150 μmol HOCl/L	-				CD	-	-			-
Yurina et al (2021) [74]	Fibrinogen +25-50-300 μmol hydrogen peroxide	-		-			=		=	=	
Yurina et al (2021) [74]	Fibrinogen +25-50-300 μmol HOCl	-		-			-		-	-	
Bettiol et al (2023) [75]	Fibrinogen + 0.01-1 mM AAPH	-	+	-	-						-
Yurina et al (2024) [56]	Fibrinogen +10μM HOCl	=		=		IF				=	=
Yurina et al (2024) [56]	Fibrinogen +25 μM HOCl	-		-		IF	-		-	+	-
Undas et al (2008) [76]	Fibrinogen from haemodialysis patients	+	-		+		-		-		-
Paton et al (2010) [77]	Fibrinogen from MI patients	+	=	+	+	PC	+			-	
Kwasny-Krochin et al (2010) [78]	Fibrinogen from RA patients	+			+		+		-		-
Becatti et al (2014) [42]	Fibrinogen from MI patients	-	+	-	-	PC-CD	-				-
Becatti et al (2016) [79]	Fibrinogen from patients with Behçet disease	-	+	-	-	PC-CD					-
Hugenholtz et al (2016) [80]	Plasma from cirrhosis patients	-		=		PC	=		-	=	
White et al (2016) [81]	Plasma from trauma patients	-						-			+
Becatti et al (2019) [82]	Fibrinogen from patients with Behçet disease	-	+	-	-		-	-		+	-
Bryk et al (2019) [83]	Fibrinogen from type 2 diabetic patients					PC			-		-
Misztal et al (2019) [84]	fibrinogen + 0–1000μM HOCl from controls					Dityr	-	-		+	-
Becatti et al (2020) [85]	Fibrinogen from cirrhosis patients	-	+	-	-	CD-Dityr-IF-PC	-	-	-	+	-
Baralic et al (2020) [86]	Fibrinogen from patients with ESRD (end stage renal disease)					CD-PC	=	=	=	=	
Kaufmanova et al (2021) [86]	Fibrinogen from patients with arterial atherothrombotic disorders	-		-			-	-	-		-
Ceznerova et al (2022) [87]	Fibrinogen from patient with thrombosis-associated hypofibrinogenemia:	-		-		CD-Dityr-IF	-			-	-
Bettiol et al (2023) [75]	Fibrinogen from GCA patients	-	+	-	-	PC	-	-	-	+	-
Błaż et al (2023) [88]	Plasma from acute ischemic stroke patients					PC	-		-	+	-
Nowak et al (2024) [89]	Plasma from atrial fibrillation patients					PC					
Słaboszewski et al (2024) [90]	Plasma from atrial fibrillation patients									+	**-**
Gitto et al (2024) [91]	Fibrinogen from liver transplant recipients	-	+	-	-	CD-Dityr-IF	-	-	-	+	-
**Glycosilation**											
Marchi et al (2004) [92]	Fibrinogen from extra glycosylated Fg patients	-	=	-	-		-	-	-	-	
Maghzal et al (2005) [93]	Fibrinogen from patients taking fibrates	-	+								
Maghzal et al (2005) [93]	Fibrinogen from pregnant women	=	=								
Morris et al (2007) [94]	Fibrinogen from chronic thromboembolic pulmonary hypertension (CTEPH) patient						-				-
Adamczyk et al (2013) [95]	Fibrinogen from healthy donors	-									
Hugenholtz et al (2016) [80]	Fibrinogen from cirrhosis patients	-				PC	=		-	=	
Gligorijević et al (2018) [96]	Fibrinogen from aging people	=		=		PC-IF	=		=		
Gligorijević et al (2018) [97]	Fibrinogen from cirrhosis patients					CD-IF-PC					
Nellenbach et al (2021) [98]	Fibrinogen from neonatal and adult patients	+ =		- =						- =	+ =
Moiseiwitsch et al (2022) [49]	Fibrinogen from COVID‐19 patients	+		+			-	+		+	-
**Glycation**											
Mirmiranpour et al (2012) [99]	Fibrinogen + 50 mM glucose	+				CD-IF					
Norton et al (2017) [100]	Fibrinogen + 5, 10, 25, or 50 mM glucose	+								+	
Hood et al (2018) [101]	Fibrinogen + 10 mMol/L glucose	+					-			+	
Rehman et al (2020) [102]	Fibrinogen + 10 mM MGO					PC-FTIR-CD-IF					
Perween et al (2019) [103]	Fibrinogen + 1.5, 7.5, 10 and 15 mM MGO					PC-FTIR-CD-IF					
Luzak et al (2020) [104]	Fibrinogen + 30mM glucose	-									
Alouffi et al (2022) [105]	Fibrinogen + 25 mM & 50 mM of glyoxal					PC-FTIR-CD-IF					
Perween et al (2022) [106]	Fibrinogen + 7.5 mM of MGO					PC-FTIR-CD-IF					
Ahmad et al (2024) [107]	Fibrinogen + 2.5, 5, 7.5, 10 mM MGO					PC-CD-IF					
Jörneskog et al (2003) [108]	Fibrinogen from type 1 diabetics								-		-
Dunn et al (2005) [109]	Fibrinogen from type 2 diabetics	+	-		+		=		-	+	
Dunn et al (2006) [94]	Fibrinogen from type 2 diabetics										-
Pieters et al (2006) [110]	Fibrinogen from type 2 diabetics	=	=	=					=	=	
Pieters et al (2008) [111]	Fibrinogen from type 2 diabetics	+					=	=	+		-
Li et al (2016) [112]	Fibrinogen from type 2 diabetics							=			
Schuett et al (2017) [113]	Fibrinogen from chronic hemodialysis patients						-		-	+	-
Luzak et al (2020) [104]	Fibrinogen from type 2 diabetics	-			=						
Rehman et al (2021) [114]	NZW female rabbits were immunized with N-Fib and MG-Fib					PC					
**Nitration**											
Gole et al (2000) [115]	Fibrinogen + 1 mM peroxynitrite	+									
Vadseth et al (2004) [44]	Fibrinogen + 100 μM nitrite		-	+		Dityr-CD					
Nowak et al (2007) [62]	Fibrinogen + 10 μM peroxynitrite	=	=	=		Dityr-PC					
Nowak et al (2007) [62]	Fibrinogen + 100 or 1000 μM peroxynitrite	-	+	-		Dityr-PC					
Ponzcek et al (2008) [116]	Fibrinogen + 10 μM NO_2_BF_4_	+	-	+							
Ponzcek et al (2008) [116]	Fibrinogen + 0.1 or 1 μM NO_2_BF_4_	-	+	-							
Bijak et al (2012) [117]	Fibrinogen + 1 μM peroxynitrite	+		+							
Bijak et al (2012) [117]	Fibrinogen + 10 μM or higher peroxynitrite	-		-			-			+	
Bijak et al (2013) [118]	Fibrinogen + 100 μM peroxynitrite	-		-							
Ding et al (2014) [119]	Bovine Fibrinogen + 8.7 μM peroxynitrite + manganese	-				IF					
Helms et al (2017) [120]	Fibrinogen +5 μM ProliNONOate	-					+	=		-	
Marchelak et al (2021) [121]	Fibrinogen + 100 μM peroxynitrite	Nakayama-Hamada et al				IF					
Rutkowska et al (2021) [122]	Fibrinogen + 100 μM peroxynitrite	+		+						+	
Farhana et al (2024) [123]	Fibrinogen + 10 μM peroxynitrite					Dityr-IF					
Vadseth et al (2004) [44]	Fibrinogen from CAD patients	+	-	+		Dityr-CD	-		-	+	=
Parastatidis et al (2007) [124]	Depletion of nitrated fibrinogen form	+									
Parastatidis et al (2008) [125]	Fibrinogen from smokers	+		+			=		+		-
Heffron et al (2009) [126]	Plasma from volunteers receinving 1 ng/kg LPS	+		=							
Nowak et al (2017) [127]	Fibrinogen from MM patients	=	+	=	=		=				-
**Citrullination**											
Nakayama-Hamada et al (2008) [128]	Fibrinogen + PAD4	-		-							
Okumura et al (2009) [129]	Fibrinogen + PAD2	-		-							
Damiana et al (2020) [130]	Fibrinogen + 0.7-20µg/ml PAD2	-		-			-			-	+(ns)
Varju et al (2021) [131]	Fibrinogen + 0.25-1,15 μg/ ml PAD4							-			**-**
Varju et al (2022) [132]	Fibrinogen + 0.25– 2.8 μg/ ml PAD4						-		-	+	
Kwasny-Krochin et al (2010) [78]	Fibrinogen from RA patients	+					-		-		-
Pretorius et al (2012) [133]	Fibrinogen from RA patients						-			+	
Vranic et al (2019) [134]	Fibrinogen from RA women						-		-	+	-
Bezuidenhout et al (2020) [135]	Fibrinogen from RA patients	+					+	-		+	=
**Acetylation**											
Antovic et al (2005) [136]	Fibrinogen +37.5 or 320 mg/day aspirin								= +		
He et al (2009) [137]	Fibrinogen + 0.04, 0.07, 0.14, 0.56, or 2.22 mmol/L of acetylsalicylic acid	=					+		+	-	+
Ajjan et al (2009) [138]	Fibrinogen + 1, 10, and 100 mg/L		-	+			+	-	+	-	+
Tehrani et al (2012) [139]	Fibrinogen from type 1 diabetics +75 or 320 mg/day aspirin								= +		
Antovic et al (2005) [136]	Plasma from voluteers taking 37.5 or 320-640 mg aspirin						+ =		+ +		
Ajjan et al (2009) [138]	Fibrinogen from voluteers taking 150 mg aspirin			=	+		+				+
**Guanidinylation**											
Schuett et al (2017) [113]	Fibrinogen +1 mol/L o-methylisourea-bisulfate						-				
Schuett et al (2017) [113]	Plasma from patients on hemodialysis						-		-		-
**Homo-cysteinylation**											
Lauricella et al (2006) [140]	Plasma + 500 μM hcys	=		+			+			+	-
Sauls et al (2006) [141]	Fibrinogen + 300 μM homocysteine thiolactone						-			+	-
Marchi et al (2008) [142]	Plasma + 13, 19, 52 μM hcys	-	+	-			=				-
Marchi et al (2008) [142]	Plasma + 251 μM hcys	=	=	=							
Marchi et al (2008) [142]	Fibrinogen + 408 μM hcys	-	+	-							
Sauls et al (2011) [143]	Fibrinogen + 300 μM homocysteine thiolactone										-
Malinowska et al (2011) [144]	Plasma + 0.1–1mM homocysteine-thiolactone	+									-
Genoud et al (2014) [145]	Fibrinogen + 100, 500 and 1,000 μmol/L homocysteine-thiolactone	-	+	-			-			+	
**Carbamylation**											
Ma et al (2021) [146]	Fibrinogen + 96 mM sodium cyanate					IF-CD					
Binder et al (2017) [147]	Fibrinogen + 5 or 100 mM KOCN	-	+	-	-		-	-		+	-
**Phosphorylation**											
Heldin et al (1987) [148]	Fibrinogen + protein kinase C			=			-				
Forsberg et al (1989) [149]	Fibrinogen + protein kinase C			-			-				
Forsberg et al (1990) [150]	Fibrinogen + protein kinase C										-
Martin and Bjork (1990) [151]	Fibrinogen + protein kinase C					CD-IF					
Martin and Bjork (1990) [151]	Fibrinogen + casein kinase II					CD-IF					
Martin et al (1991) [152]	Fibrinogen + protein kinase A			-			-				-
Martin et al (1991) [152]	Fibrinogen + protein kinase C			-			-				
Martin et al (1991) [152]	Fibrinogen + casein kinase I			=			=				-
Martin et al (1991) [152]	Fibrinogen + casein kinase II			+			-				-
Suk et al (1997) [153]	Fibrinogen + casein kinase II	+		+							
Martin et al (1992) [154]	Fibrinogen from patients after hip surgery			+			+				-
Reganon et al (1993) [155]	Fibrinogen from patient’s acute myocardial infarction			+							-
**Dephosphorylation**											
Forsberg et al (1989) [149]	Fibrinogen + alkaline phosphatase			+			+				
Forsberg et al (1990) [150]	Fibrinogen + alkaline phosphatase										=
Martin et al (1991) [152]	Fibrinogen + alkaline phosphatase			+			+				
Martin et al (1992) [154]	Fibrinogen + alkaline phosphatase			+			+				

*PC* Protein carbonyls, *CD*Circular dichroism, *IF* Intrinsic fluorescence,*FTIR* Fourier transform infrared spectroscopy, *Dityr* Dityrosine crosslinking

The table summarizes findings from multiple studies analyzing the effects of different PTMs on fibrinogen polymerization, denotes an increase, "-" denotes a decrease, "=" denotes no change

**Table 2:** Summary of the effects of fibrinogen PTMs on fibrin clot function

**Modifications**	**Involved Sites**	**Functional Effects**
**Acetylation**	Aα: K191, K208, K224, K429, K457, K523, K539 Bβ: K122, K130, K133, K217, K233, K234, K318, K323, K344 γ: K170, K273	= Rate of polymerization - Resistance to fibrinolysis + Diameter of fibers + Permeability - Density
**Amidation**	Aα: Y43 Bβ: Y142, Y152, Y225, Y239, Y285, Y292, Y326 γ: Y96, Y109	Unknown
**Carbamylation**	Unknown sites	- Rate of polymerization - Resistance to fibrinolysis - Diameter of fibers + Density
**Citrullination**	Aα: R16, R19, S22, R50, R65, R95, R104, R110, R116, R141, R159, R162, R167, R197, R199, R244, R252, R268, R289, R334, R348, R375, R385, R406, R407, R424, R439, R440, R491, R493, R528, R554, Y570 Bβ: R14, R17, R23, R30, R42, R44, R94, R101, R104, R194, R216, R255, R346, R380, R391, R406, R415, R455 γ:R5, R14, R34, R45, R108, R197, R256, R391	- Rate of polymerization = Resistance to fibrinolysis - Diameter of fibers - Permeability + Density
**Dichlorination**	Aα: unknown sites Bβ: Y285, P289 γ: Y96, Y109	Unknown
**Glycation**	Aα: K52, K81, R95, R110, R141, R167, R197, R199, K208, R244, R268, K413, K448, R491, R493, R528, K539, R554, K562, R572 Bβ: R23, R91, K122, K133, K148, R166, R169, R176, K181, R194, K209, K217, K283, R304, K323, R380, R406 γ: K62, K85, K120, K140, K151, K212	+/= Rate of polymerization + Resistance to fibrinolysis -/= Diameter of fibers + Density Structural alterations described
**Glycosylation**	Aα: T320, S351, N453, N686 Bβ: N364, N394 γ: N52, N78, K85, N334	- Rate of polymerization -/= Diameter of fibers Structural alterations described
**Guanidinylation**	Unknown sites	- Diameter of fibers - Permeability
**Hydroxylation**	Aα: K508, P546, P565 Bβ: P31 γ: unknown sites	Unknown
**Homocysteinylation**	Aα: K52, K70, K81, H138, K191, K230, K413, K418, K448, K562 Bβ: K181, K217, K298, K344, K396 γ: K85, K95, K170, W266, K273, K373, K385	= Rate of polymerization + Resistance to fibrinolysis + Density
**Malonylation**	Aα: unknown sites Bβ: S184, C394, S395 γ: unknown sites	Unknown
**Methylation**	Aα: D56, E179, N277, D477, E520, E578 Bβ: E25, K134, E141, E147, D154, E220, D240, E245, E334, D383, D389 γ: E13, E72, D80, E97, E213	Unknown
**Nitration**	Aα: Y43, Y76, Y258, Y570 Bβ: Y41, Y119, Y142, Y192, Y285, T292, Y345, Y378, Y417, T422, Y445 γ: T96, Y109, Y114, T262, T274, T348,T349, Y354, T363	Low level of nitration: + Rate of polymerization High level of nitration: - Rate of polymerization +/- Resistance to fibrinolysis Structural alterations described
**Oxidation**	Aα: H24, D32, W33, P34, K70, H84, M91, H132, N139, R141, M147, K148, K176, Y178, H201, M207, K208, P209, D212, K219, W229, D234, M235, M238, M240, M255, Y258, W276, N287, N288, W302, P304, P309, W315, W341, W391, S432, H456, P473, M476, D477, H492, H494, K508, F513, F514, P516, M517, E526, R528, H544, H545, P546, Y570, M584, D586, H594, H598 Bβ: H16, Y41, R42, H67, F115, Y117, M118, Y119, K122, K133, Y142, H149, Y152, D154, M190, P196, K217, M224, M242, N246, W249, W266, F273, P289, Y292, W293, M305, P307, M314, W317, K318, H325, Y338, M367, H370, H371, M373, M376, D381, D383, W385, W402, W403, Y417, W419, Y422, W424, M426, H429, W437, M438, W440, K441, W444, Y445, M447, K453, F458 γ: K58, K62, Y68, P70, K75, P76, N77, M78, D80, M89, M94, K95, H103, K120, K125, K140, W208, F215, H217, P220, F226, W227, K232, H234, Y244, W253, M264, Y274, F322, H340, H343, N345, W372, R375, K380, K381, H400, H401, K406	- Rate of polymerization + Resistance to fibrinolysis - Diameter of fibers - Stiffness of clot - Permeability + Density Structural alterations described
**Phosphorylation**	Aα: S3, S22, S45, S50, S56, S259, T268, S272, W275, S279, S281, S291, S294, S297, S299, S345, S364, S365, T393, T412, S436, S441, S451, S485, S486, S489, T505, T522, S523, S524, S542, S546, S551, S557, S558, S559, S560, S561, S572, S585, S590, S594, S599 Bβ: S67, S173 γ: S68, Y389, T400, S404, T416, S420	- Resistance to fibrinolysis - Diameter of fibers Structural alterations described
**Sulfation**	Aα: unknown sites Bβ: unknown sites γ: Y418, Y422, Y444, Y448	+ Rate of polymerization

This table lists specific PTMs, the corresponding amino acid sites on fibrinogen chains where these modifications occur, and the resultant functional effects on clot formation and degradation. Symbols indicate the type of functional change observed: '+' denotes an increase, '-' denotes a decrease, and '=' indicates no change. Each letter-number combination indicates the type of amino acid, represented by its one-letter code (e.g., *A* for Alanine, *R* for Arginine, *N* for Asparagine, *D* for Aspartic acid, *C* for Cysteine, *Q* for Glutamine, *E* for Glutamic acid, *G* for Glycine, *H* for Histidine, *I* for Isoleucine, *L* for Leucine, *K* for Lysine, *M* for Methionine, *F* for Phenylalanine, *P* for Proline, *S* for Serine, *T* for Threonine, *W* for Tryptophan, *Y* for Tyrosine, *V* for Valine), and its position within the chain

### Techniques for studying protein modifications and conformational changes

PMTs at different molecule sites can significantly alter fibrinogen structure and therefore its functional properties. Thus, the analysis of fibrinogen structural alterations is crucial to give information about possible biological effects of PTMs.

Fibrinogen PTMs and structural alterations can be investigated by:

- Mass spectrometry (MS) currently represents the gold standard method and the most informative technique for protein PTMs analysis.
- Circular Dichroism (CD) spectroscopy is used to investigate the protein secondary structure. CD protein spectra in the far ultraviolet (UV) range (180–260 nm) depends on the electronic excitation of the partially delocalized peptide bonds, which form the backbone of the polypeptide chain. Therefore, this method detects changes in the main alpha-helical peptide backbone structure [73]. Moreover, the CD spectra in the near ultraviolet (UV) range (250–350 nm) is used for the analysis of protein tertiary structure.
- Fourier-transform infrared (FTIR) spectroscopy provides information about protein secondary structure. FTIR spectroscopy functions by exposing a sample to infrared radiation to determine which wavelengths are absorbed within the infrared spectrum. Each compound exhibits a distinctive pattern of absorption bands in its infrared spectrum. (Characteristic bands find in the infrared spectra of proteins and polypeptides include Amide I and Amide II)
- X-ray crystallography and Nuclear Magnetic Resonance (NMR) spectroscopy provide information about protein tertiary structure.
- Fluorescence spectroscopy is a powerful technique widely employed in the study of protein structure due to its sensitivity and versatility. By exciting proteins with ultraviolet or visible light, fluorescence spectroscopy can provide valuable insights into their structural characteristics and environment. The emission spectra generated reveal information about the protein tertiary structure, such as the presence and accessibility of tryptophan, tyrosine, and phenylalanine residues, which are intrinsic fluorophores in proteins. Changes in fluorescence intensity, wavelength, or polarization can indicate alterations in protein conformation or interactions with ligands, cofactors, or other proteins. Moreover, fluorescence spectroscopy can be employed in both steady-state and time-resolved modes, allowing researchers to probe dynamics, folding kinetics, and stability of proteins under various conditions.

Fibrinogen oxidation has been extensively studied, revealing several significant impacts on its structure and function. Numerous observations demonstrate that fibrinogen oxidation results in (1) important changes in the secondary structure of fibrinogen that are manifested in diminishing the alpha-helical content [42, 68, 79, 91, 156, 157] (2) chemical transformation of highly susceptible methionine residues [53–56, 81] and other sulphur containing side chains as well as of cyclical aminoacid residues [42, 68, 79, 156, 157]; (3) dose-dependent increase in side chain carbonyl group content [42, 79, 88–90, 157, 158]; (4) dityrosine crosslinks formation by tyrosine residues oxidation [157, 159, 160]; (5) reduction of aliphatic CH_2_ and CH_3_ moieties [161].

Peroxynitrite (ONOO^−^) is an oxidant and a nitrating agent capable of oxidizing cysteine and tryptophan residues. The exposure of fibrinogen to peroxynitrite in vitro causes nitrative/oxidative modifications [47, 62, 121, 123, 162] and ONOO^−^-induced modification of fibrinogen has been found to result in the formation of 3-nitrotyrosine, dityrosine crosslinking and carbonylation [163]. Parastatidis et al. [125] and Hoffman et al. [164] reported elevated levels of 3-nitrotyrosine in fibrinogen from cardiovascular disease patients, indicating a prothrombotic risk factor.

In contrast, Vadseth et al. [44] demonstrated that alterations in the properties of fibrinogen and fibrin clots following treatment with nitrating agents occur without dityrosine cross-linking or changes in fibrinogen secondary structure, as assessed by CD spectroscopy.

To explore the impact of hyperglycosylation on fibrinogen structure, several studies have been conducted. Far-UV CD spectra of fibrinogen revealed a reduction in the α-helix content in fibrinogen originating from patients with cirrhosis compared to the healthy controls. Near-UV CD spectra showed slight differences between the two groups, suggesting a possible change in the protein tertiary structure [97]. Spectrofluorimetric analysis revealed a reduction in the intrinsic fluorescence of fibrinogen from the patients, confirming that its tryptophan residues resided in the altered surrounding. All these data are in line with those observed for fibrinogen oxidation [97]. Also, Hugenholtz et al. [80] showed a significantly increased fibrinogen carbonyl content in the same condition. Conversely, in the context of aging, which is associated with increased protein oxidation, the level of protein carbonyls in healthy older individuals was not significantly higher compared to younger individuals, although changes in the tertiary structure of fibrinogen were observed [96].

Some studies [103, 106, 107, 114] showed that in vitro treatment with methylglyoxal (MGO) resulted in fibrinogen structural and conformational changes. The formation of fibrinogen-advanced glycation end products (AGEs) compromised the functional properties of fibrinogen. Fluorescence, FTIR, and CD results indicate that glycation impacts both the secondary and tertiary structure of fibrinogen [102, 105]. Similar findings were reported by Mirmiranpour et al. [99], where the CD spectra showed changes in both the secondary and tertiary structures of fibrinogen after glycation, including a reduction in the α-helical content.

In vitro experiments on phosphorylation showed that fibrinogen phosphorylated by both protein kinase C (PKC) and casein kinase 2 (CK2) underwent a conformational change in their secondary structure. Conversely, phosphorylation by protein kinase A (PKA) or protein kinase C(PKC) induced changes in the tertiary structure of fibrinogen, particularly around tryptophan residues [151].

Fibrinogen PTMs such as amidation, dichlorination, hydroxylation, malonylation, methylation and sulphation have been described, but the effects on fibrinogen structure are unknown [47, 57].

## PTMs and fibrin clot architecture

Fibrin clot architecture, characterized by fiber diameter and pore size within the fibrin network, is critical for its biological function in hemostasis, fibrinolysis, and wound healing [165]. The impact of PTMs on clot properties can be evaluated by measuring fibrin fiber diameter, clot stiffness, clot permeability, clot density and cross-linking, which involves covalent cross-links between fibrin α and γ chains.

### Effects of oxidation

Oxidation represents the most extensively studied fibrinogen PTM. It occurs when ROS are produced excessively and not neutralized by antioxidants. External factors like radiation, drugs, and pollution can also increase ROS levels, leading to oxidative stress, which damages biological macromolecules, including DNA, proteins, and lipids, causing mutations, loss of function, and cellular damage [18].

In vitro studies using various oxidation protocols (e.g., irradiation, photooxidation, ozone, ascorbate/FeCl_3_, peroxynitrite, HOCl, glycolaldehyde) have shown conflicting results regarding fibrin fiber diameter, with most studies reporting smaller diameters [53, 55, 56, 63, 68–70, 73, 75, 76, 78, 80, 82, 84–88, 91, 166] while only a few studies (one in vitro using ozone as oxidant condition, and two ex vivo) report different results [66, 77, 78].

Other characteristics, such as reduced stiffness [53, 55, 61, 70, 73, 81, 82, 84–86, 166], lower permeability [53, 56, 72, 75, 76, 78, 80, 83, 85, 88, 91, 166], increased fibrin clots density [53, 55, 56, 68–70, 72, 75, 82, 84–86, 88, 90, 91] and an enhanced cross-linking [63, 70, 84] have been observed with oxidized fibrinogen.

### Effects of nitration

Nitration, another significant PTM, primarily affects tyrosine and cysteine residues, forming 3-nitrotyrosine and 3-nitrocysteine. This modification is usually driven by neutrophils and monocytes, which produce nitrating agents in inflammatory sites and venous thrombi [47, 126].

Fibrinogen nitration has been studied in a few cases, producing conflicting results likely due to varying levels of nitration. Some studies reported significantly smaller fibrin fiber diameter [44, 117], while others found no change [125, 127] or even an increase [120]. However, other clot properties, such as stiffness and rigidity [44, 120, 125], density [117, 120, 122], permeability [44], and cross-linking [44, 127] were generally consistent with expectations: thinner fibers led to denser, less permeable clots.

### Effects of glycosylation and glycation

Glycosylation, the covalent attachment of carbohydrate to protein during biosynthesis via N-glycosidic or O-glycosidic bonds, includes sialylation, where sialic acid is the terminal monosaccharide.

Studies evaluating the role of glycosylation and sialylation showed mixed results: one study [92] found reduced fibrin fiber diameter, stiffness, permeability and density, while two others [80, 96] observed no changes. Hypersialylation, on the other hand, was found to produce clots with thinner fibers, greater stiffness and increased density.

Glycation, a non-enzymatic reaction between a lysine residue’s ε-amino group and a sugar molecule’s aldehyde group, is common in diabetes due to hyperglycaemia. Following glycation, fibrin fiber diameter and clot stiffness were either unchanged [109, 111, 112] or decreased [101, 113], while three out of five studies reported decreased permeability [108–111, 113] and generally increased density [100, 101, 109, 110, 113]. Only one study reported no difference in cross-linking between fibrinogen from patients with diabetes mellitus and control subjects [111].

### Effects of acetylation and phosphorylation

Acetylation of fibrinogen, particularly in the context of aspirin treatment, modifies several lysine residues: Aα (K191, K208, K224, K429, K457, K523, K539); Bβ (K233), and γ (K170, K273), resulting in increased fibrin fibers diameter, higher permeability, reduced clot density, and lower stiffness [136–139].

The effects of acetylation vary with aspirin dosage: low doses enhance fiber mass/length ratio and permeability, while higher doses have little impact on fiber thickness but slightly increase permeability, especially in type 1 diabetes patients due to reduced acetylation of glycated fibrinogen [167].

Phosphorylation, a reversible process mediated by a serine/threonine or tyrosine protein kinase, regulates fibrinogen’s clot-forming properties by altering fiber diameter: phosphorylation by PKA or PKC reduces fibrin fiber diameter [149, 152, 168], while phosphorylation by CK2 increases it [152]. Accordingly, experiments involving dephosphorylation demonstrate an increase in fiber diameter [149, 152, 154].

In a study by Martin et al. [154], increased fibrinogen phosphorylation during the acute phase following hip-replacement surgery was associated with thicker fibrin fibers. These findings suggest that casein kinase II may play a significant role in ex vivo fibrinogen phosphorylation.

### Effects of homocysteinylation, citrullination, and other PTMs

Fibrinogen homocysteinylation, involving the acylation of ε-amino group of lysine residues by homocysteine thiolactone or the oxidation of cysteine thiol groups, occurs with elevated plasma homocysteine levels. Studies on fibrinogen homocysteinylation [140–145] have reported conflicting effects on fibrin fiber diameter, with reports of no change [142], increases [140] or decreases [141, 145], depending on homocysteine concentrations and the experimental conditions (e.g., plasma vs. purified fibrinogen). Most studies observed increased clot density [140, 141, 145], but permeability and stiffness were not widely evaluated.

Citrullination, the enzymatic conversion of arginine to citrulline by peptidylarginine deiminase (PAD) [130], consistently leads to a reduction in fibrin fibers diameter [78, 130, 132–134], decreased permeability [78, 132, 134] and denser clots [131–135].

Other fibrinogen PTMs, such as carbamylation, results in thinner fibers, increased clot density, and reduced cross-linking [147], while guanidinylation [113] produces clot with thinner fibers and decreased permeability.

Fibrinogen PTMs such as amidation, dichlorination, hydroxylation, malonylation, methylation and sulphation have been described, but the effects on fibrin clot architecture are unknown [47, 57].

## PTMs and clot formation

During coagulation, thrombin cleaves fibrinogen, releasing FPA and FPB from the N-termini of the Aα- and Bβ-chains, converting fibrinogen to fibrin monomers. Insertion of these newly exposed α- and β- “knobs” into a- and b- “holes” in the γC and βC regions of the D nodule, respectively, on another fibrin monomer permits the half-staggered association of fibrin monomers into protofibrils. Subsequent aggregation of protofibrils into fibers, through lateral aggregation promoted mainly by intermolecular αC: αC interactions and probably also by interactions between both α- and γ-chains, yields a fibrin network that is essential for blood clot stability [33, 129, 165, 169, 170].

The effects of PTMs on fibrinogen can significantly impact clot formation kinetics, which can be evaluated by measuring four key parameters: (i) thrombin-catalyzed fibrin polymerization, which assesses the conversion of fibrinogen to fibrin and determines clotting time or aggregation rate; (ii) maximum velocity (V max), indicating the speed of lateral protofibril association; (iii) lag phase, indicating the time until fibril aggregation begins; and (iv) maximum turbidity or absorbance (MaxAbs) of the clot, reflecting the final clot structure in terms of fibrin fiber size and protofibril density [85].

### Effects of oxidation

Fibrinogen oxidation is a critical post-translational modification that can significantly alter the process of fibrin formation and clot dynamics. Most studies consistently report that fibrinogen oxidation significantly reduces its conversion to fibrin compared to non-oxidized fibrinogen (Table 1). The lag phase is consistently prolonged across nearly all experiments [42, 62–64, 66, 75, 79, 82, 85], while the maximum absorbance and maximum velocity, measured in turbidity assays, are consistently decreased [42, 56, 58, 62–65, 67, 68, 72, 74, 75, 82, 85, 87, 91, 166]. However, the effects of oxidation on fibrin clot architecture are not uniform across all studies. Variations in experimental conditions, such as different concentrations of oxidizing agents, and differences in patient populations contribute to conflicting findings regarding polymerization rates and clot characteristics. Torbitz et al. [52] and several ex vivo investigations [76–78] have shown an increased polymerization rate. The in vitro study by Torbitz et al. used relatively high concentrations of HOCl (1, 2, 4 mM), potentially explaining this deviation from other findings [52]. Ex vivo studies examining patients with end-stage renal disease on hemodialysis, myocardial infarction (MI), and rheumatoid arthritis (RA) have yielded conflicting results. For instance, Undas et al. [76] observed significant differences in the lag phase among hemodialysis patients compared to controls, whereas patients on peritoneal dialysis exhibited a higher rate of protofibril formation in another study [171], possibly due to elevated fibrinogen levels in these subjects. Similarly, Paton et al. showed [77] higher polymerization rate and increased maximum turbidity in oxidized fibrinogen from MI patients. In contrast, Becatti et al. [42] observed a slower rate of thrombin-catalyzed fibrinogen polymerization in patients with post-acute MI (6 months after the event). This discrepancy could be attributed to differences in the patient cohorts enrolled in the studies.

Kwasny-Krochin et al. [78] conducted the first study on fibrin clot structure/function in RA patients, revealing faster but less permeable and poorly lysable fibrin clots, due to elevated acute phase proteins such as fibrinogen and C reactive protein (CRP) during active disease phases. Salonen and coworkers [172], provided a mechanistic link by showing that CRP binds to fibrinogen and fibrin, potentially influencing fibrin clot structure under pathological conditions.

In summary, while fibrinogen oxidation generally reduces fibrin formation and alters clot characteristics, the specific effects on clot architecture and polymerization dynamics vary significantly depending on the oxidizing conditions, experimental setups, and patient characteristics, underscoring the complexity of fibrinogen’s role in different pathological states.

### Effects of nitration

Studies on fibrinogen nitration, particularly ex vivo experiments involving patients with coronary artery disease, smokers, healthy volunteers taking lipopolysaccharides and patients with multiple myeloma (MM), consistently show higher levels of fibrinogen nitration compared to controls. Generally, nitrated fibrinogen demonstrates an increased conversion rate to fibrin, which aligns with shorter lag phases and higher maximum absorbance in turbidity assays, indicating an accelerated polymerization process [44, 124–126]. However, the effects of nitration on fibrin clot appears to be concentration dependent. Low concentrations of peroxynitrite (< 10 µM) typically show an enhanced fibrin formation [115–117, 122], while higher concentrations of nitration agents (> 10 µmol/L peroxynitrite or 100 µmol/L nitronium fluoroborate) typically reduce polymerization rate [62, 118–120]. For instance, Ding et al. [119] observed a decreased polymerization rate when using 8.7 µM peroxynitrite in combination with increasing manganese levels, which enhances nitration. Helms et al. found a longer clotting time and decreased initial rate of clot formation with 5 µmol/L ProliNONOate, a nitric oxide donor, although these results were not statistically significant. Conversely, some studies have reported increased polymerization rates despite high peroxynitrite concentrations, which could be due to relatively low levels of nitration or the presence of only a few modified fibrinogen molecules, as suggested by Gole et al. [115], de Vries [32], Rutkowska [122] and Vadseth [44]. This variability underscores the complexity of nitration effects on fibrinogen and the need to consider the specific nitration conditions in interpreting the results.

### Effects of glycosylation and glycation

As aging is associated with increased fibrinogen glycosylation, but Gligorijević et al. found no significant differences in clotting speed and maximal fibrin clot optical density across different age groups [96]. Other studies found that the extra carbohydrate moiety impairs the protofibril lateral association process, resulting in a decreased polymerization rate [80, 92, 95]. As for fibrinogen sialylation, a reduced conversion into fibrin and an increase in lag phase was reported in hepatoma, liver disease and fibrate therapy patients [93, 173–175].

Nellenbach et al. [98] demonstrated that hypersyalilation in neonates increases fibrin polymerization rate, but these effects disappear when sialic acid was removed.

Moiseiwitsch et al. [49] showed that COVID-19 patients have higher sialic acid content in fibrinogen, leading to faster polymerization and greater maximum turbidity, which is responsible for the altered clot density in these patients.

Regarding glycation, most studies showed an increased polymerization rate when fibrinogen was incubated with glucose [99–101, 109, 111], while only one study reported a decreased rate compared to control [104]. This reduction was attributed to glycation’s effect on fibrinogen clotting ability, which involves the formation of strong covalent bonds and the influence of elevated glucose concentrations during fibrin polymerization, resulting in weaker interactions and a reduced maximal velocity of fibrin polymerization in diabetic patients.

### Effects of acetylation and phosphorylation

Fibrinogen acetylation has significant effects on clotting dynamics and clot structural properties, with older in vitro studies [176–180] showing reduced maximum turbidity of fibrin polymerization in the presence of high doses of aspirin or acetylating agents. However, more recent studies have reported increased or unchanged turbidity values [137, 138]. Acetylation generally impairs fibrinogen clotting property, making fibrin fibers thicker, leading to a looser network in a dose-dependent manner.

In terms of phosphorylation, several protein kinases, including PKA, PKC, and CK1 and CK2 [181–184], can phosphorylate fibrinogen, altering clot properties. CK2-dependent fibrinogen phosphorylation increases clot turbidity and significantly enhances the rate of blood coagulation in vitro [153, 185], while PKC-dependent fibrinogen phosphorylation reduces clot turbidity [148–150, 168]. These effects are further confirmed by studies on fibrinogen dephosphorylation with alkaline phosphatase [149, 150, 152, 154]. Ex vivo studies have reported that increased fibrinogen phosphorylation following hip surgery or myocardial infarction (MI) leads to faster polymerization rates [154, 155].

### Effects of homocysteinylation, citrullination, and other PTMs

Homocysteinylation, evaluated in vitro by incubating fibrinogen or plasma with different concentrations of homocysteine, has shown mixed effects on clotting ability, with some studies reporting decreased polymerization rate, reduced maximum turbidity, and a prolonged lag phase, while others reported contradictory findings [140, 142, 144, 145].

Citrullination, studied in vitro with PAD2 and PAD4 enzymes, inhibits fibrin polymerization by preventing thrombin-catalyzed release of fibrinopeptides [128–130]. Ex vivo studies [78, 135] in rheumatoid arthritis patients demonstrated increased fibrin citrullination in plasma, leading to faster polymerization rates compared to controls.

Carbamylation, a non-enzymatic PTM resulting from the reactions with isocyanic acid [186, 187], is more common in patients with chronic kidney disease or inflammatory conditions and is linked to impaired fibrin clot formation [188]. I*n vitro* studies have shown that carbamylation reduces fibrinogen conversion to fibrin, lowering maximum turbidity and velocity, while increasing lag phase [146, 147].

Tyrosine sulfation has been suggested to play important roles in blood coagulation and it is responsible for facilitating key protein–protein interactions. In addition, it has been described that sulfation of fibrinogen enhances binding affinity to thrombin, increasing the rate of polymerization [189–192].

Fibrinogen PTMs such as amidation, dichlorination, hydroxylation, malonylation and methylation have been described, but the effects on fibrin clot formation are unknown [47, 57].

## PTMs and clot lysis

The fibrinolytic system plays a crucial role in maintaining haemostatic balance by breaking down fibrin, the final product of blood coagulation, through the action of plasmin [165, 193].

Fibrinogen PTMs can significantly influence not only clot formation but also clot lysis, thereby impacting the overall process of fibrinolysis. Various studies have explored the effects of different PTMs on fibrin degradation, revealing that can alter clot lysis in diverse ways [42, 53, 63, 67, 71, 73, 75, 76, 78, 79, 82–85, 87, 166]. These modifications can either decrease fibrinolytic activity, as seen with oxidation and phosphorylation, or enhance clot degradation, as observed with certain carbohydrate modifications and acetylation. The impact of these PTMs on fibrinolysis is complex and varies depending on the specific type of modification, the conditions under which it occurs, and the presence of additional factors such as disease states or therapeutic interventions.

Oxidation significantly impacts fibrinogen structural integrity and fibrin susceptibility to plasmin-induced lysis. Several studies highlight that fibrinogen oxidation led to a decreased fibrinolytic activity. This is evident from the impaired clot dissolution observed in inflammatory conditions such as Bechet’s disease, where neutrophil activation promotes fibrinogen oxidation, resulting in resistant thrombus formation [79]. Similarly, patients with pulmonary hypertension exhibit increased fibrinogen oxidation, which correlates with reduced plasmin-mediated fibrin degradation [194]. In Giant Cell Arteritis (GCA), a chronic inflammatory disease affecting large and medium-sized arteries, the risk of thrombosis is significantly elevated due to a combination of vascular inflammation, endothelial dysfunction, and increased oxidative stress. This oxidative stress promotes fibrinogen oxidation, altering its structure and function, leading to the formation of denser, more resistant fibrin clots [75]. The oxidative stress-related structural changes include increased dityrosine cross-linking and altered tertiary structure, which collectively reduce the fibrin clot susceptibility to plasmin-mediated lysis [42]. Moreover, anti-inflammatory interventions, such as IL-6 inhibition with tocilizumab, have been shown to restore redox balance and partially reverse the oxidation-induced fibrinogen modifications, thereby enhancing fibrinolytic efficiency in affected patients [75]. On the contrary, in the study by White et al. [81], fibrin polymerization was found to be impaired in trauma patients with increased fibrinogen Aα-Met476(SO), leading to decreased clot strength and increased fibrinolysis after injury.

Overall, these findings underscore the critical role of oxidative stress in modulating fibrinogen function and clot lysis, emphasizing the need for targeted therapies to mitigate oxidative damage in thrombotic disorders.

Nitration is another PTM that has been studied for its impact on fibrin clot degradation, primarily through ex vivo experiments conducted in patients with coronary artery disease (CAD), Multiple Myeloma (MM) and smokers. The results have been somewhat mixed, with one study showing no significant difference in fibrinolysis respect to control, while two studies demonstrated a decrease in fibrinolysis [44, 125, 127].

The modification of fibrinogen by carbohydrates, particularly through glycation and hypersialylation, has also been investigated, albeit in a limited number of studies.

Glycation, commonly occurring in patients with diabetes mellitus or those undergoing chronic hemodialysis, has consistently been shown to reduce fibrinolytic activity across four different studies [94, 108, 111, 113]. Conversely, the effects of hypersialylation on fibrinolysis appear more variable. Among three studies examining this PTM, two reported a decrease in clot degradation, while one observed an increase [49, 98, 195]. Specifically, Moiseiwitsch et al. [49] investigated fibrinogen from COVID-19 patients, finding it to have a higher content of sialic acid residues compared to controls. The removal of these residues led to a significant increase in the rate of clot degradation, highlighting the influence of hypersialylation on fibrin stability.

As previously discussed, aspirin-induced fibrinogen acetylation is another PTM that alters clot structure and function. This modification results in a less compact fibrin network, which shortens the lysis time of clots formed from aspirin-treated fibrinogen. These findings have been supported by both in vitro and ex vivo studies [137, 138].

Fibrinogen phosphorylation has been consistently associated with a reduction in fibrin degradation, regardless of the kinase involved [150, 152, 154, 155]. Interestingly, when fibrinogen is dephosphorylated using alkaline phosphatase, clot degradation is not affected, suggesting that phosphorylation specifically contributes to the resistance of fibrin to plasmin-induced lysis [150].

Additional PTMs, such as homocysteinylation, carbamylation and guanidinylation have similarly been associated with decreased fibrin degradation. However, the effects of citrullination on clot degradation are less clear, with studies showing conflicting results [78, 113, 130, 134, 135, 140–144, 147].

Fibrinogen PTMs such as amidation, dichlorination, hydroxylation, malonylation, methylation and sulphation have been described, but the effects on fibrin clot lysis are unknown [47, 57].

## Conclusions

**Fig. 3:**
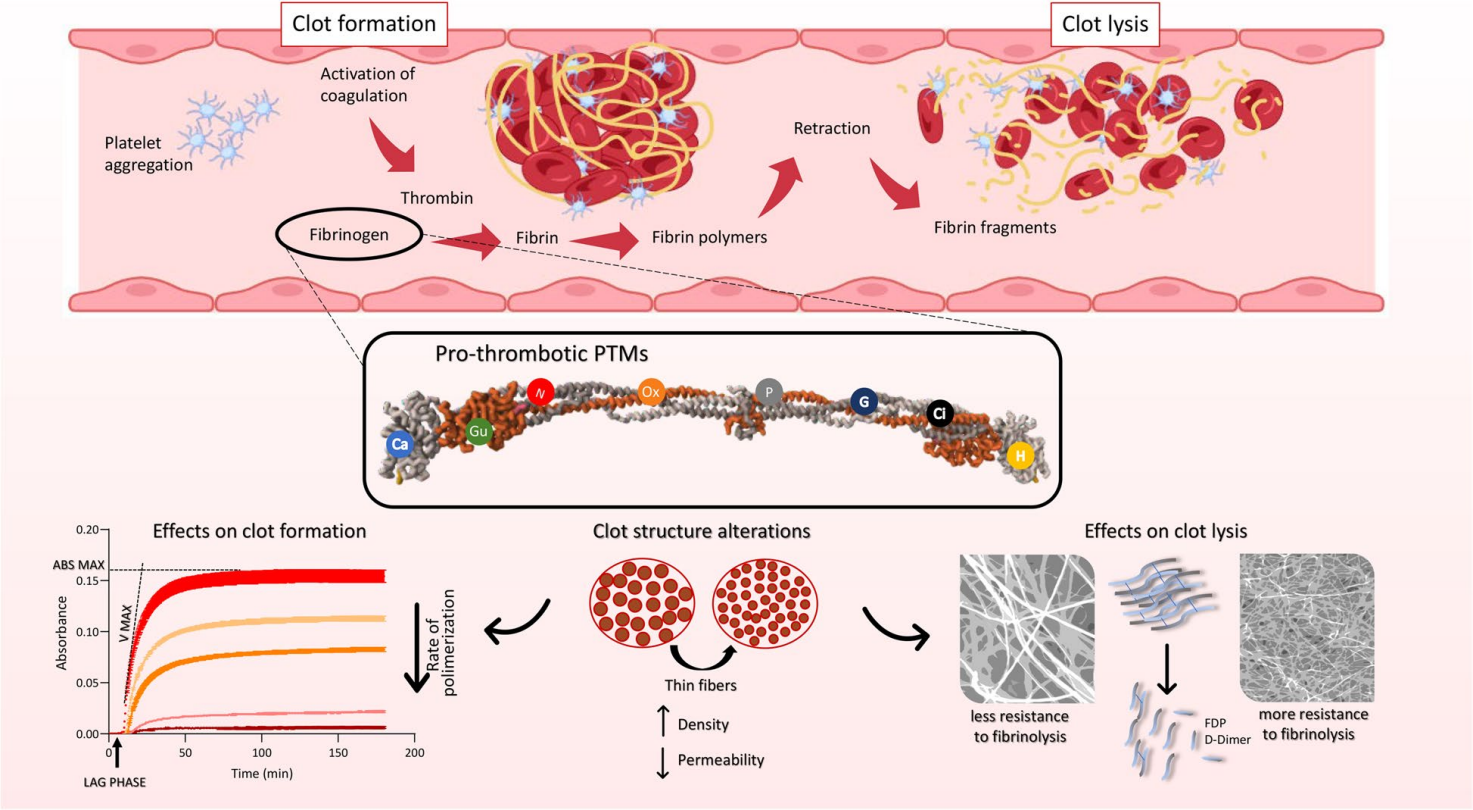
Impact of PTMs on fibrin clot properties The top panel (created with BioRender.com) illustrates the stages of clot formation and clot lysis, starting with platelet aggregation and activation of the coagulation cascade, leading to the conversion of fibrinogen into fibrin, and eventually the degradation of the clot into fibrin fragments The central panel highlights the role of pro-thrombotic PTMs on the fibrinogen molecule, depicting various modifications that alter fibrinogen’s structural properties (Ox (Oxidation), N (Nitration), P (Phosphorylation), G (Glycation), Gu (Guanidinylation), Ca (Carbamylation), H (Homocysteinylation), S (Sulfation), Ac (Acetylation), and M (Methylation) The bottom panel displays the effects of PTMs on clot formation parameters, such as lag phase, maximum absorbance, and maximum velocity. It also shows how PTMs result in clot structure alterations, including thinner fibrin fibers, increased clot density, and reduced permeability, which affect the clot’s susceptibility to fibrinolysis leading to thrombosis complications

An increasing body of research indicates a connection between thromboembolic events and distinct prothrombotic structural features of fibrin clots. Our review highlights that fibrinogen PTMs, such as oxidation, nitration, glycosylation, glycation, acetylation, phosphorylation, and others, significantly influence the biochemical and mechanical properties of fibrin clots. These modifications can alter clot architecture by affecting fibrin polymerization rates, fiber thickness, clot density, and susceptibility to fibrinolytic degradation, ultimately modulating thrombus stability and resolution (Fig. 3).

Oxidation and nitration typically lead to denser clots with thinner fibers, reducing clot permeability and increasing resistance to fibrinolysis, which can exacerbate prothrombotic conditions such as cardiovascular diseases, chronic inflammatory disorders, and diabetes mellitus, especially under high oxidative stress. Conversely, modifications like acetylation, often induced through aspirin therapy, result in more permeable clots with thicker fibers, enhancing fibrinolytic susceptibility. This highlights the therapeutic potential of aspirin and other antiplatelet drugs in reducing thrombotic risk and managing conditions such as coronary artery disease and stroke.

The effects of other modifications, such as glycation and phosphorylation, are particularly relevant in the context of metabolic disorders like diabetes, where elevated glucose levels lead to increased glycation of fibrinogen, further complicating the thrombotic profile of these patients. The modulation of these PTMs presents an opportunity for pharmacological intervention aimed at altering clot properties to favor fibrinolysis and reduce thrombus formation. Such strategies could include antioxidant therapies to reduce oxidative stress, which is known to promote fibrinogen oxidation, or the use of specific inhibitors that target detrimental PTMs without disrupting beneficial ones.

It is important to note that approximately 70% of the studies reviewed here were performed in vitro, using varying concentrations of chemicals to induce PTMs. While in vivo studies are limited primarily to the major modifications, they generally corroborate the effects observed in vitro. However, few studies have investigated other modifications, necessitating further validation.

Despite the increasing number of studies on fibrinogen modifications, few have identified site-specific modifications and linked them to molecule function and in vivo effects. Therefore, additional experimental and clinical investigations are essential to pinpoint PTMs sites *in vivo.* Studies employing human fibrinogen, where feasible, will be crucial in understanding how these site-specific modifications affect function and protein interactions. While significant progress has been made in understanding the effects of PTMs on fibrinogen structure, clot formation, and fibrin degradation, it is crucial to acknowledge that many limitations and gaps still exist in this area of research. One of the primary challenges in studying fibrinogen PTMs is the variability in experimental conditions, which can lead to discrepancies in results across different studies. Moreover, the diversity of PTMs detection methods and the absence of standardized protocols complicate the direct comparison of findings. Additionally, differences in patient populations and physiological conditions introduce further variability, making it difficult to isolate the specific impact of each PTM. Future research should aim to address these challenges by developing standardized methodologies and exploring the effects of PTMs in more diverse and clinically relevant settings. Expanding our understanding of these modifications could provide insights into their broader implications in thrombosis and other coagulation disorders, ultimately informing the development of targeted therapeutic strategies.

## Data Availability

Not applicable.

## Abbreviations

CK: Casein Kinase
CD: Circular Dichroism
FPA: Fibrinopeptide A
FPB: Fibrinopeptide B
FTIR: Fourier-Transform Infrared (Spectroscopy)
MI: Myocardial Infarction
MM: Multiple Myeloma
PAD: Peptidylarginine Deiminase
PK: Protein Kinase
PTMs: Post-Translational Modifications
RA: Rheumatoid Arthritis
ROS: Reactive Oxygen Species
